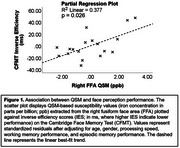# Reduced Face Perception in Aging Linked to Iron Accumulation in Face‐Processing Brain Regions

**DOI:** 10.1002/alz70857_099903

**Published:** 2025-12-24

**Authors:** Valentinos Zachariou, Merlene Behrmann, Yang Jiang, Barbara S Nikolajczyk, Philip A Kern, Elif PINAR Coskun

**Affiliations:** ^1^ University of Kentucky, Lexington, KY, USA; ^2^ University of Pittsburgh, Pittsburgh, PA, USA; ^3^ University of Kentucky, SBCoA, Lexington, KY, USA

## Abstract

**Background:**

Face perception is fundamental for human social interactions, with declines in this ability linked to increased social anxiety, impaired social interactions, and reduced quality of life. For older adults, who are particularly vulnerable to social isolation—a known risk factor for dementia—face perception is especially critical. However, face perception deteriorates earlier and independently of general cognitive decline, increasing the risk of adverse functional outcomes. Our goal is to elucidate mechanisms that give rise to this deterioration. Our recent longitudinal findings reveal localized age‐related iron accumulation in specific cortical regions, including key face‐processing areas in inferior temporal cortex. Given iron's role as both essential for brain health and a potent oxidizer capable of damaging neurons and myelin, we hypothesize that age‐related iron accumulation in face‐processing regions contributes to face perception decline.

**Methods:**

Eighteen healthy older adults (65‐78 years old) with corrected vision underwent structural MRI, quantitative susceptibility mapping to assess brain iron, and functional MRI during a faces vs houses task to identify face‐processing regions (e.g. the fusiform face area; FFA) and control regions (e.g. the house‐selective parahippocampal place area; PPA). Behavioral performance was assessed using the Cambridge Face and Car Memory Tests (CFMT/CCMT) with the car category as a control. Linear regression analyses assessed relationships between iron concentration in localized regions and CFMT/CCMT performance, controlling for age, gender, and NIH Toolbox measures of processing speed, working memory, and episodic memory.

**Results:**

Higher iron concentration in the right FFA significantly correlated with lower CFMT performance (inverse efficiency scores: higher scores = lower performance; *p* =  0.026; *r* ^2^ = 0.377; β = 1.06; t = 2.58; Figure 1). No significant associations were observed between FFA iron and CCMT performance (*p* = 0.104) or between PPA iron and CFMT performance (*p* = 0.960). Processing speed, working memory, and episodic memory were not significantly associated with CFMT scores (*p* > 0.19).

**Conclusion:**

These preliminary findings suggest that higher iron levels in face‐processing regions are specifically associated with declines in face perception, independent of general cognitive performance. Further research is needed to assess how brain iron impacts the functional and white‐matter connectivity of brain networks supporting face perception.